# Spatial disaggregation of tick occurrence and ecology at a local scale as a preliminary step for spatial surveillance of tick-borne diseases: general framework and health implications in Belgium

**DOI:** 10.1186/1756-3305-6-190

**Published:** 2013-06-22

**Authors:** Valerie Obsomer, Marc Wirtgen, Annick Linden, Edwin Claerebout, Paul Heyman, Dieter Heylen, Maxime Madder, Jo Maris, Maude Lebrun, Wesley Tack, Laetitia Lempereur, Thierry Hance, Georges Van Impe

**Affiliations:** 1 Université Catholique de Louvain, Earth and Life Institute, Georges Lemaitre climate and earth research centre, place Louis Pasteur 3, 1348, Louvain la Neuve, Belgium; 2 Biodiversity department ELIB, Université Catholique de Louvain, Earth and Life Institute, 4 place Croix du sud, 1348, Louvain-la-Neuve, Belgium; 3 Department of infectious and parasitic diseases, health and pathology of the wildlife, University of Liège, boulevard de Colonster 20, 4000, Liège 1, Belgium; 4 Laboratory of Parasitology, Faculty of Veterinary Medicine, Ghent University, Salisburylaan 133 9820, Merelbeke, Belgium; 5 Reference Laboratory for Vector-Borne Diseases, Queen Astrid Military Hospital, Bruynstraat 1, 1120, Brussels, Belgium; 6 Evolutionary ecology group, Department of Biology, University of Antwerp, Groenenborgerlaan, 171-2020, Antwerpen, Belgium; 7 Institute for Tropical Medicine, 155 nationalestraat, B2000, Antwerpen, Belgium; 8 Department of Veterinary Tropical Diseases, University of Pretoria, Pretoria, South Africa; 9 ARSIA, Allée des Artisans, 2 - 5590, Ciney, Belgium; 10 Dierenarts Gezondheidszorg Herkauwers Veepeiler, DGZ Vlaanderen, l Hagenbroeksesteenweg 167 l, 2500, Lier, Belgium; 11 Laboratory of Forestry, Department of Forest and Water Management, Ghent University, Geraardsbergsesteenweg 267, 9090, Melle-Gontrode, Ghent, Belgium; 12 Laboratory of Parasitology and Pathology of Parasitic Diseases, Department of Infectious and Parasitic Diseases, Faculty of Veterinary Medicine, University of Liège, Bd de Colonster 20 B43, 4000, Liège, Belgium

**Keywords:** Tick, Vector, Spatial distribution, Ecology, Vector-borne diseases

## Abstract

**Background:**

The incidence of tick-borne diseases is increasing in Europe. Sub national information on tick distribution, ecology and vector status is often lacking. However, precise location of infection risk can lead to better targeted prevention measures, surveillance and control.

**Methods:**

In this context, the current paper compiled geolocated tick occurrences in Belgium, a country where tick-borne disease has received little attention, in order to highlight the potential value of spatial approaches and draw some recommendations for future research priorities.

**Results:**

Mapping of 89,289 ticks over 654 sites revealed that ticks such as *Ixodes ricinus* and *Ixodes hexagonus* are largely present while *Dermacentor reticulatus* has a patchy distribution. Suspected hot spots of tick diversity might favor pathogen exchanges and suspected hot spots of *I. ricinus* abundance might increase human-vector contact locally. This underlines the necessity to map pathogens and ticks in detail. While *I. ricinus* is the main vector, *I. hexagonus* is a vector and reservoir of *Borrelia burgdorferi s.l.,* which is active the whole year and is also found in urban settings. This and other nidiculous species bite humans less frequently, but seem to harbour pathogens. Their role in maintaining a pathogenic cycle within the wildlife merits investigation as they might facilitate transmission to humans if co-occurring with *I. ricinus*. Many micro-organisms are found abroad in tick species present in Belgium. Most have not been recorded locally but have not been searched for. Some are transmitted directly at the time of the bite, suggesting promotion of tick avoidance additionally to tick removal.

**Conclusion:**

This countrywide approach to tick-borne diseases has helped delineate recommendations for future research priorities necessary to design public health policies aimed at spatially integrating the major components of the ecological cycle of tick-borne diseases. A systematic survey of tick species and associated pathogens is called for in Europe, as well as better characterisation of species interaction in the ecology of tick-borne diseases, those being all tick species, pathogens, hosts and other species which might play a role in tick-borne diseases complex ecosystems.

## Background

The incidence of tick-borne diseases is increasing in Europe [1] and follows an increase in the number of tick bites [2] attributed to two factors: abundance of questing ticks and human exposure to ticks [3]. Measures targeting human exposure by promoting timely removal of ticks failed to stop the rise in Lyme borreliosis incidence in the Netherlands. On the other hand, this rise was related to an increase in *Ixodes ricinus* abundance [3]. Knowing the local variations in the distribution of the species interacting in tick-borne diseases systems, including ticks, pathogens and species influencing the presence and abundance of ticks and pathogens, could provide new opportunities to estimate potential infection risks locally, identify local hot spots and develop targeted prevention, surveillance and control.

Necessary information is lacking at national and sub national levels in many countries. The first missing information concerns the presence and distribution of tick species. Efforts to characterise tick distribution on a European scale [4-6] are limited by the information available at sub national level and only target major vectors such as *I. ricinus*. Other tick species less willingly biting humans sometimes harbour high pathogen prevalence’s and might contribute locally to the pathogens’ cycles [7]. The role of all tick species present should be investigated jointly per pathogen and their distribution clarified. The second missing information concerns the spatial distribution of hosts, predators and species influencing tick populations and pathogens’ prevalence in ticks. The presence and abundance of tick species varies locally according to many factors, including host availability [3]. Pathogen prevalence in ticks also varies locally according to availability of reservoirs, dead-end hosts and vectors [8]. The third set of missing information concerns pathogens associated to ticks, their presence, reservoirs, vectors and distribution. Pathogens found using classical PCR methods are those searched for, while others might be present but undetected. Because micro-organisms are increasingly found in ticks, a more systematic approach is needed. The list of micro-organisms found locally or abroad in local tick species could be narrowed by clarifying pathogenicity, vector capacity and presence of reservoirs to provide a list of potential pathogens to investigate locally. This would clarify the spectrum of pathogens potentially transmitted locally through a tick bite.

In this paper, occurrence records and information on tick species relevant for public health have been compiled for a country where tick-borne diseases received little attention. In Belgium, the limited quality of current information is obvious because of proximity to the Netherlands, a country that stands out for efficient investigations of tick-borne diseases. In the Netherlands, tick bites are subject to spatial monitoring [9]. Lyme borreliosis is monitored by physician surveys targeting *Erythema migrans*, the most common symptom [2]. In Belgium, only Lyme borreliosis is regularly diagnosed. In 2009, official numbers of cases varied from 500 to 1500 [10] or 9000 cases [11] according to the source. In the Netherlands, 22000 cases were recorded for that same year [2]. *I. ricinus* is believed to occur in Southeast Belgium but records occur elsewhere. Detailed distribution can approximate local exposure to ticks. This is of direct public health interest because according to European guidelines for Lyme borreliosis, an individual presenting an *Erythema migrans* is considered a confirmed case if potentially exposed to areas favourable for vector ticks [12]. Laboratory confirmation and remembering a tick bite are not necessary to confirm this diagnosis.

This study aims to show how an integrated spatial approach on tick species in a given country can provide the preliminary information needed for adapted national public health policies by providing: 1) a list of tick species present and their detailed distribution, 2) the micro-organisms they could harbour, 3) ecological traits influencing vector status, 4) implications for public health and suggestions for future research priorities.

## Methods

Three sources of tick locations were considered: new tick collections, literature collections and “grey datasets”. Quality levels are proposed for each record to document for example accuracy of tick location according to type of host/vegetation (lower if captured on moving animals such as dog or deer). Information on localisation, collection, vegetation or host, pathogen load, and original data source were compiled in an excel database (Additional file 1). A systematic literature search was made based on ISI web of knowledge using the keywords “tick AND ecology” from 1989 to 2001 and “tick” from 2002 to 2011. Additional articles resulted from a specific search on presence and pathogenicity of microorganisms found in ticks. The database and additional literature articles form the basis for this paper and are examined for the following items: geographical distribution, species behaviour, ecology, presence of micro-organisms. Consequences for public health and prevention are highlighted. For some sites additional details were provided by authors, however, the methodology is already described in published articles and summarised in the Additional file 1. This includes (1) Collection BAYER, 579 sites [13], (2) Collection RLVBD, 51 sites [14], (3) Collection UGENT FOREST, 33 sites [15,16], (4) Collection ARSIA, 17 sites [17], (5) Collection UCLIREC, 5 sites [18], and (6) Collection UA1, 16 sites [7,19-22]. Collection GREY DATA includes tick field observations from the website of NATAGORA and NATUURPUNT (http://www.observations.be, http://www.waarnemingen.be) by registered users involved with nature related activities from 1980 until February 2012. Methodologies for original tick collections are described in detail below:

### Collection VANIMPE

In the framework of the convention 5284a funded by IRSIA (Institut pour l′encouragement de la recherche dans l′industrie et l′agriculture), the center of acarology (UCL) led two collection campaigns in the Campine, the plateau brabançon and the Condroz. The regions were selected based on local Lyme borreliosis cases and favourable tick habitat. The first campaign in 1989 targeted 30 sites and the second campaign prospected 234 sites from May to October 1990 including 79 days of prospection. Ticks were collected from the environment by flagging. Each collection lasted 2 hours and UTM coordinates were checked on maps (100 m).

### Collection WILDSCREEN

From 2007 to 2009, ticks from wild cervids (*Cervus elaphus* and *Capreolus capreolus*) found dead, hunted or killed for sanitary reasons were collected by the Wild Screen Network disease monitoring activities in Southern Belgium [23]. Ticks were preserved in 70% ethanol at room temperature, and morphologically identified up to stage and species level (by L. Lempereur and A. Nahayo). Sex and repletion were recorded. *Dermacentor reticulatus* was also collected on wild cervids from 2010 to 2012. As *D. reticulatus* and *D. marginatus* may show overlapping phenotypes [24], a PCR was used for confirmation, targeting the *Dermacentor* second Internal Transcribed Spacer 2 (ITS2) with the following primers: ITS_forward (5′-GTG-CGT-CCG-TCG-ACT-CGT-TTT-GA-3′) and ITS_reverse (5′-ACG-GCG-GAC-TAC-GAC-GGA-ATG-C-3′) [25]. The DNA purification was carried out using the NucleoSpin tissue kit for tissue protocol (Macherey-Nagel GmbH, Germany). Samples were frozen in liquid nitrogen and homogenized on a Tissue Lyser^®^ (Qiagen, GmbH, Germany). PCR conditions were as follow: each reaction was carried out in 50 μL volume containing 4 μl of the DNA preparation, 5 μl of each 2 mM dNTP, 2 μl of each 10 μM oligonucleotide primer, 2 U of TaqDNA polymerase (New England Bio labs) with 5 μl of the 10x PCR supplied buffer and completed to 50 μl with sterile water. PCR was achieved with an initial denaturation cycle at 95°C for 5 min, followed by 35 cycles (94°C, 45 s), annealing (53°C, 45 s), extension (72°C, 70 s) and a final extension step at 72°C for 10 min. All ITS2 PCR products were sequenced using a modified Sanger method with the Big Dye terminator kit version 3.1 and resolved with a 3730 ABI capillary sequencer (Applied Bio systems). Sequencing reaction was performed with the same primers as for the PCR and sequences aligned by BLAST search.

### Collection ITG

Ticks were collected by flagging for several years (site 1, 60, 172).

### Collection IRSNB

Additional tick locations registered in museum collections were provided by the IRSNB (Institut Royal des Sciences Naturelles de Belgique).

### Collection DGZ

While investigating anaplasmosis in 11 farms in Flanders, in 2011, the Dierengezonheidzorg (DGZ – Animal Health Care Flanders, Belgium) recorded *I. ricinus* ticks on several animals from those 11 farms.

### Collection GLAXOSMITHKLINE

Ticks were captured by flagging in 1999 in 3 sites in Belgium (sites 228, 242, 353).

### Collection MARTIN

*D. reticulatus* was found on a human around Namur and a trypanosome discovered in the intestine of *I. ricinus* in the context of other research [26].

### Collection UGENT VETE

The clinic of poultry diseases of Ghent University performs diagnosis required by individuals. In this framework they recorded a tick infestation on a pigeon from *Argas* species, probably *Argas reflexus* in July 2012 near Berlaar (site 303) in the province of Antwerp.

## Results and discussion

**Figure 1:**
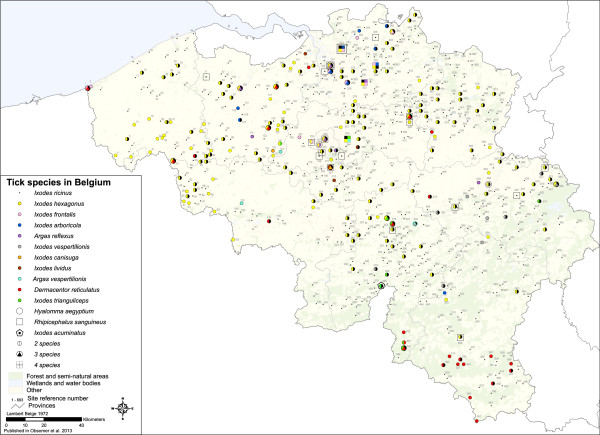
**Map of tick species recorded in Belgium before 2011. **Map of existing tick species records in Belgium. One color corresponds to one species. When two or more species occur at the same site special symbols compile the colors of the two or more species. *I. ricinus* is in black color. Numbers labelling each site correspond to site number in Additional file 1.

The database includes 1624 records for a total of 89,289 ticks and is summarized per collection in Table 1. Records within 3 km range were grouped for the display in 654 sites. Original details are available for each record (Additional file 1) and can be visualised using Google Earth (Additional file 2). Figure 1 shows sites of occurrence for all tick species.

**Table 1:** Tick species found in Belgium per collection

	**Species**	**Number of species**	**Number of ticks**	**Number of records**
	*Ixodes ricinus *(1801/395)			
	*Ixodes hexagonus *(634/164)			
	*Dermacentor reticulatus *(18/5)			
Collection BAYER	*Rhipicephalus sanguineus *(6/5)	4	2264	577
	*Ixodes ricinus *(868/271)			
	*Dermacentor reticulatus *(297/10)			
Collection GREY DATA	*Ixodes lividus *(5/1)	3	1170	282
	*Ixodes ricinus *(43150/175)			
	*Ixodes lividus *(7/2)			
	*Ixodes hexagonus *(792/33)			
	*Rhipicephalus sanguineus *(97/13)			
	*Argas reflexus *(17/7)			
	*Argas vespertilionis *(9/3)			
	*Hyalomma aegyptium *(26/4)			
	*Ixodes acuminatus *(1/1)			
	*Ixodes arboricola *(190/3)			
	I*xodes canisuga *(2/1)			
	*Ixodes frontalis *(7/4)			
	*Ixodes trianguliceps *(9/8)			
Collection LITERATURE	*Ixodes vespertilionis *(29/11)	13	44,655	273
	*Ixodes ricinus *(5819/192)			
	*Ixodes hexagonus *(1/1)			
Collection VANIMPE	*Rhipicephalus sanguineus *(1/1)	3	5821	194
	*Ixodes ricinus *(2232/87)			
Collection WILDSCREEN	*Dermacentor reticulatus *(159/11)	2	2391	98
	*Ixodes ricinus *(4000/8)			
	*Dermacentor reticulatus *(66/66)			
Collection ITG	*Ixodes frontalis *(2/2)	3	4068	76
Collection RLVBD	*Ixodes ricinus *(22435/45)	1	22,435	45
	*Ixodes ricinus *(2670/5)			
	*Ixodes hexagonus *(1/1)			
	*Ixodes arboricola *(2790/12)			
Collection UA	*Ixodes lividus *(18/2)	4	5571	29
Collection ARSIA	*Ixodes ricinus* (600/17)	1	600	17
	*Ixodes ricinus *(6/4)			
	*Ixodes hexagonus *(4/2)			
	*Dermacentor reticulatus *(2/1)			
	*Argas vespertilionis *(5/1)			
	*Dermacentor reticulatus *(2/1)			
	*Ixodes canisuga *(1/1)			
	*Ixodes frontalis *(1/1)			
	*Ixodes trianguliceps *(1/1)			
Collection IRSNB	*Ixodes vespertilionis *(18/4)	9	38	15
Collection DGZ	*Ixodes ricinus *(11/11)	1	11	11
Collection GLAXOSMITHKLINE	*Ixodes ricinus *(167/3)	1	167	3
	*Ixodes ricinus *(1/1)			
Collection MARTIN	*Dermacentor reticulatus *(1/1)	2	2	2
Collection UGENT	*Argas reflexus *(1/1)	1	1	1
TOTAL		14	89289	1624

Species collected (number of ticks per species / number of location records per species), number of species, number of ticks and number of location recorded per collection. When the number of collected ticks is unknown the value is set to 1. (Details in Additional file 1).

### Tick species in Belgium

Fourteen species were recorded in Belgium and 10 additional species are suspected to be present because they were recorded in surrounding areas [27-32] (Table 2). *I. ricinus* (Linnaeus, 1758) and *I. hexagonus* (Leach 1815) are widely distributed (Figure 1). *I. ricinus* is found in all the provinces. *I. hexagonus* is also present in all provinces and is known to be more abundant than *I. ricinus* in specific settings such as urban gardens [33]. These species are also the easiest to catch through flagging (*I. ricinus*) or because they are present on pets investigated by veterinarian surveys. Other species records are sporadic and difficult to interpret. Those records might reflect patchy distribution for ticks associated with bat habitats for example, or just lack of systematic surveys for the recently investigated *D. reticulatus* (Fabricius, 1794) [34]. The distribution of species with 20 or fewer occurrences is difficult to analyse. Areas without records are due to absence or absence of surveys, thus leading to difficulties in the interpretation of the data.

**Table 2:** Tick species of Belgium

**Tick name**	**Records in BE (Ticks)**	**Year for last record**	**NL**	**NW EU**	**N FR**	**GE**
**Established in the wild**						
*Ixodes ricinus*	1223 (88758)	2011	Yes	Yes	Yes	Yes
*Ixodes hexagonus*	201(1333)	1987	Yes	Yes	Yes	Yes
*Dermacentor reticulatus*	102 (861)	2012	Yes	Yes	Yes	Yes
*Ixodes frontalis*	16 (102)	2011	Yes	Yes		Yes
*Ixodes arboricola*	15(2980)	2011	Yes	Yes		Yes
*Ixodes lividus*	5 (30)	1989	Yes	Yes		Yes
*Ixodes canisuga*	2 (3)	1945	Yes	Yes		Yes
*Ixodes trianguliceps*	9 (10)	1987	Yes	Yes		Yes
*Ixodes acuminatus*	1 (1)	1987		Yes		
*Argas reflexus*	8 (18)	2012	Yes	Yes		Yes
*Argas vespertilionis*	4 (14)	1942	Yes	Yes		Yes
*Ixodes vespertilionis*	15 (47)	1969	Yes	Yes		Yes
**Established in houses and recurrently imported on host**						
*Rhipicephalus sanguineus*	19 (104)	1982	Yes	Yes	Yes	Yes
**Not established but recurrently imported on host**						
*Hyalomma aegyptium*	4 (26)	(1965)	Yes	Yes		
**Potentially present but never found in Belgium**						
*Ixodes unicavatus*	Potential	Never	Yes	Yes	Maybe	No
*Ixodes uriae*	Potential	Never		Yes	Yes	Yes
*Ixodes ventalloi*	Potential	Never		Yes	Yes	
*Ixodes apronophorus*	Potential	Never	Yes	Yes (UK)		Yes
*Ixodes rugicollis*	Potential	Never			Yes	
*Ixodes simplex*	Potential	Never				Yes
*Dermacentor marginatus*	Potential	(1973)		Yes	Yes	Yes
*Haemaphysalis punctata*	Potential	Never	Yes	Yes (UK)	Yes	Yes
*Haemaphysalis concinna*	Potential	Never		Yes	Yes	Yes
*Haemaphysalis inermis*	Potential	Never		Yes	Yes	

List of tick species in Belgium (BE), the Netherlands (NL), North Western Europe (NW EU) including the United Kingdom (UK), North France (N FR) and Germany (GE). Number of records in BE (Total numbers collected include available records for larvae, nymphs and adult stages). Some collectors, however, do not collect the larvae even if they are present. The year of the last records is in brackets if only found on hosts from import. Other species are present in surrounding countries but were not considered because they are very rare (*Rhipicephalus bursa*, *Amblyoma variegatum*) or specific to host species rare in Belgium (*Ixodes unicavatus*, *Ixodes rotchildi*, *Ixodes caledonicus*, *Ornithodoros maritimus*).

Most tick species are nidiculous and thus found mainly on hosts, nests or burrows. Off host habitats are important for *I. ricinus* and *D. reticulatus* which quest on the vegetation. *I. ricinus* is found in deciduous forests, pastures bordered by trees, hedges and bushes [35], or with tall grass and high humidity [27], vegetated sand dunes [3], pine forests [14] and city parks [36]. Contradictory definitions of *D. reticulatus* habitats exist in the literature [32] with *D. reticulatus* found in drier areas than *I. ricinus*[37] or in moist areas along rivers [38] or on the fringe of meadows in dry vegetation [39] and variation in abundance in similar sites [40]. Many vertebrates are parasitized by *I. ricinus* (Table 3). *I. hexagonus* is present on many mammals but never birds [39]. Other species seem restricted to specific hosts such as birds (*I. frontalis* (Panzer, 1798), *I. arboricola* Schulze & Schlottke, 1930, *Argas reflexus* (Fabricius, 1794), *I. lividus* Koch, 1844), rodents (*I. trianguliceps* Birula, 1895, *I. acuminatus* Neumann, 1901), mammals (*I. canisuga* Johnston, 1849*, D. reticulatus*) or bats (*Argas vespertilionis* (Latreille, 1796), *I. vespertilionis* Koch, 1844). *Rhipicephalus sanguineus* (Latreille, 1806) is imported on dogs and found occasionally in houses. *Hyalomma aegyptium* (Linnaeus, 1758) is imported on tortoises. Names are such as revised by Barker and Murrell [41] or used by Petney *el al*. [32]. While seasonality might be linked to host life cycle for bat ticks, most nidiculous ticks seem active year round (Table 4). *I. ricinus* is more frequently recorded in spring and autumn but the decrease in summer seems to be an artefact due to vegetation stages influencing flagging efficiency [42]. Slight activity occurs in winter. *D. reticulatus* seems mainly absent on the vegetation in summer.

**Table 3:** **Tick hosts and ecology in Belgium (details in Additional file****2****)**

	**host**	**off hostOOff**
*I. ricinus*	*Canis lupus familiaris, Felis silvestris catus, Erinaceus europaeus, Bos taurus, Homo sapiens, Capreolus capreolus, Carduelis chloris, Cervus elaphus, Parus major, Cyanistes caeruleus, Anthus pratensis, Anthus trivialis, Apodemus sylvaticus, Clethrionomys glareolus, Erithacus rubecula, Hippolais icterina, Sturnus vulgaris, Talpea europaea, Turdus ericetorum, Phylloscopus erolius, Turdus pilaris, Turdus merula, Phylloscopus inornatus, Turdus iliacus, Sitta europea, Ficedula hypoleuca, Fringilla coelebs, Lacerta vivipara, Bubo bubo*	Plant species*: Fagus sylvatica, Carpinus betulus(hornbeam), Betula pendula (birch), Quercus robur (oak), Quercus petraea (oak), Castanea sativa, Anemone nemorosa, Convallaria majalis, Prunus padus, Pteridium aquilinum, Athyrium filix-femina, Calamagrostis epigejos, Calluna vulgaris, Cytisus scoparius, Dryopteris filix-mas, Sorbus aucuparia, Cytisus scoparius, Holcus lanatus, Holcus mollis, Juncus effusus, Molinia caerulea, Persicaria hydropiper, Urtica dioica, Acer pseudoplatanus, Convallaria majalis, Maianthemum bifolium, Carpinus* sp*.., Corylus avellana, Cerasus sp, Sambucus nigra,Crataegus monogyna, Vaccinium myrtillus / Pinus, Hedera helix, Rubus fructicosus, Quercus robur & Carpinus* sp*., Molinia caerulea*
		Soils: loam or silt with limestone, clay and limestone or schists, leaf litter, schist in Famenne, limestone from Givet, sandstone, poor acid sandy soils, siliceous rock, nettles, impermeable clay soils
		Habitat: grazed pasture, forest ecotone, mixed acidophilous to acidophilous oak stands, birch stand with eagle fern, grassy path, garden, urban parcs, forest, dense thicket of beech, forest secondary pine poor acid sandy soils
*D. reticulatus*	*Capreolus capreolus, Cervus elaphus, Homo sapiens, Canis lupus familiaris*	Plant species: g*rasses, hawthorn, blackthorn (Prunus spinosa), brambles blackberry (Rubus fruticosus), birch (Betula pendula), mixture of grasses, hornbeam (Carpinus betulus), woodland (mainly Picea abies), ferns (Pteridium aquilinum), jennets (Genista scorpius), oak (Quercus robur)*
		Habitat: Fallow land, marshland, pasture used for grazing, woodland open
*I. hexagonus*	*Felis silvestris catus, Canis lupus familiaris, Ericaneus europeus, Cervus elaphus, mustela putorius*	Rabbit burrow, in herbis, in grassy nest, in house, burrow of *Meles meles*, endolithe nest of *Coloeus monedula*, pasture with edges or forest, impermeable clay soils, cave, burrow of fox
*I. canisuga*	*Polecat: Mustela putorius*	Riparian nest
*I. trianguliceps*	*Rodents, Rattus rattus, Rattus norvegicus, Apodemus sylvaticus, Clethrionomys glareolus*	Burrow of rodents
*I. acuminatus*	*Rodents: Apodemus sylvaticus*	
*I. frontalis*	*Birds: Parus major, Turdus merula, Sylvia atricapilla, Cyanistes caeruleus, Sturnus vulgaris, Parus montanus, Turdus viscivoru*	It is sometimes found in understorey vegetation, experimental nest box
*I. arboricola*	*Birds: Parus major, Cyanistes caeruleus, Sitta europea, Corvus monedula*	Occurs in particular in bird nests inside cavities (like tree-holes for example), nest, Delichon urbica nest, experimental nest box
*I. lividus*	*Birds: Riparia riparia*	Riparia riparia (nest)
*R. sanguineus*	*Canis lupus familiaris*	house
*A. reflexus*	*Columba livia*	flat, house, dovecot
*A.. vespertilionis*	*Bats: Pipistrellus pipistrellus, Eptesicus serotinus, rhinocephalus hipposideros*	
*I. vespertilionis*	*Bats: Rhinolophus hipposideros, Rhinolophus ferrumequinum, Barbastelle, Myotis myotis*	Cave wall and on stalagmites
*H.aegyptium*	*Tortoise: Testudo graeca, Testudo mauritanica*	

**Table 4:** Seasonality of tick observations (number of ticks) in Belgium

	**Jan**	**Feb**	**Mar**	**Apr**	**May**	**Jun**	**Jul**	**Aug**	**Sep**	**Oct**	**Nov**	**Dec**
*I. ricinus*	2	22	1008	851	2333	1288	1979	1559	978	948	158	60
*D. reticulatus*	150	12	1	2				1	1	38	117	2
*I. hexagonus*	4		2	13	9	32	13	13		1	3	
*I. canisuga*			1	2								
*I. trianguliceps*	1		1		1	2				2	2	
*I. acuminatus*					1							
*I. frontalis*					1			10	5	1	1	
*I. arboricola*	1	1				196					1	
*I. lividus*			3		13		14					
*R. sanguineus*					6			49				
*A. reflexus*						5	100	1	1		1	
*A. vespertilionis*				4								3
*I. vespertilionis*	5	4	11	2		1	8		2			
*H. aegyptium*					22	2					2	

Co-occurrence of tick species in some sites raises the question of potential interactions with species recorded together on the same hosts [33], or their eggs found in the same shelter [43,44]. Sites with high diversity of tick species might be hot spots of potential micro-organism exchanges. Indeed, although ticks specific, for example, to birds rarely bite humans, they might maintain a cycle of pathogens in their host populations. Those might be picked up by the generalist species *I. ricinus*, and passed onto humans [7,45]. The possibility that co-occurring exotic and local species might facilitate establishment of exotic micro-organisms should be investigated.

### Microorganisms associated to tick species

In addition to potential paralysis caused by the saliva of some female ticks, which seems very rare in Europe [46], the main impact of ticks on human health is through transmission of pathogens. Ticks acquire microorganisms through an infected meal or transovarial transmission. Micro-organisms recorded in ticks might come from a recent blood meal and presence in a tick does not mean that this tick species is a competent vector. For Ixodidae ticks feeding once per stage, the microorganisms need to survive molting and be transmitted to the next host while argasidae nymphs and adults bite repeatedly. Then, to be a pathogenic for humans, they must cause symptoms in humans. A list of 300 recorded micro-organism/tick associations is presented in Additional file 3. Some sources have a low reliability but this exhaustive list is a basis for systematic investigations and reliability of vector status and pathogenicity are compiled to propose priorities for investigations. Associations are recorded mostly outside Belgium as this was little investigated in the country. Notably, while mycoplasmas are increasingly related to ticks in the USA [47], and their prevalence is increasing throughout Europe [48,49], there are no investigations of *Mycoplasma* in Belgian tick species in the literature.

**Figure 2:**
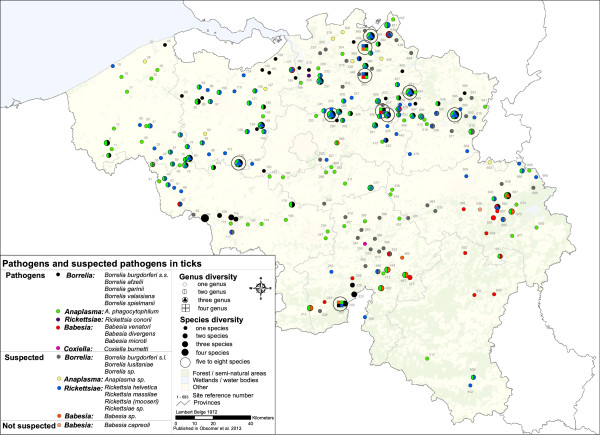
**Map of the micro-organisms, suspected pathogens and pathogens found in ticks of Belgium. **Map of existing information on micro-organisms found in ticks in Belgium. Information is presented at the genus level but if available, details of species are provided in additional file 2. Numbers labelling each site correspond to site number in Additional file 1.

For ticks present in Belgium, more than 100 associations with tick-borne (suspected) pathogens have been documented (Table 5) with some recorded in Belgium (Figure 2). Pathogenic *Borrelia* (*Borrelia burgdorferi* s.s*., B. valaisana, B. garinii, B. afzelii, B. spielmanii*), *Anaplasma phagocythophilum* and suspected pathogens such as *Borrelia lusitaniae* and *Rickettsia* (*R. helvetica*, *Rickettsia* sp., *R. massilae)* seem to be present throughout the country. Transovarial transmission of some *Babesia* species including *Babesia divergens*[50], *B venatorum*[51], (but not *B. microti*) [52] occurs in *I. ricinus*. A *Babesia* belt goes from Couvin (site 212) to Verviers (site 667) and has been documented for many years [53]. Other sites are based on ticks found on dogs and the place of infection is unsure, but a local focus of *Babesia canis* exists near Mons [54]. Some sites show a high diversity with more than 5 (suspected) pathogens. In site 575 (Genk), 481 (Ham) and 271 (Boortmeerbeek), high diversity might be linked to the presence of many ticks from many hosts. This means that (1) those localities have a higher diversity of (suspected) pathogens or (2) diversity is high everywhere but picked up there because of abundant tick material, or (3) diversity is high when ticks are abundant. Site 575 presents five pathogens (*Borreliae burgdorferi* s.s*., B. valaisiana, B. afzelii, B. garinii* and *A. phagocytophilum*) and three suspected pathogens (*B. lusitaniae*, *Rickettsia helvetica and Rickettsia* sp*.*) found in ticks collected from 38 cats (quite sedentary), thus suggesting a potential local transmission. Most interestingly, 4 pathogens (*A. phagocytophilum*, *B. burgdorferi* s.s*., B. afzelii, B. garinii*) and two suspected pathogens (*Babesia* sp*.*, *Anaplasma* sp.) were recorded from unfed *I. ricinus* nymphs (135 ticks) during flagging at site 297 (Vierves-sur-Viroin), highlighting vector status and local presence of the pathogens. This area seems to be a hot spot of *I. ricinus* abundance and the only place with up to 6 potential pathogens recorded in unfed questing ticks. Site 64 (Chercq) stands out with 5 cats harbouring 60 *I. ricinus* but no pathogens.

**Table 5:** Tick/micro-organism associations for which pathogenic status and vector status for human should be investigated as a priority: pathogens and suspected pathogens/tick species associations found abroad or in Belgium referenced in the literature for tick species found in Belgium

**Tick species**	**V/U**	***Borrelia***	***Babesia***	***Rickettsia***	***Coxiella, Franciscella Anaplasma***	**Virus**	**Other**
*I. ricinus*	V	burgdorferi sensu stricto*, afzelii*, garinii*, lusitaniae valaisiana*, spielmanii*, sp bavariensis*, miyamotoi*,	venatorum*, divergens*, microti*	***helvetica***	F. tularensis* A. phagocytophilum*	CCHF*, TBEV*, Louping Ill*	
	U	*ruskii*	*bovis, bigemina, rodhaini*	prowazekii*, conorii*,slovaca*, monacensis*, felis*, massiliae*, typhi*, sp.	C. burnetii*	WNV*, Eyack*, Erve* Tettnang*, Tribec*, KEMV*	Bartonella henselae*, Serratia marcescens*, Staphylococcus aureus*, Candidatus Neoehrlichia mikurensis*, Toxoplasma gondii*, Pasteurella pneumotropica*, Chromobacterium violaceum*, Pseudomonas aeruginosa*, Diplorickettsia massilinsis
*I. frontalis*	U	afzelii*, garinii*, turdi-like burgdorferi sensu lato,			C. burnetii*	KEMV*, TBEV*	Candidatus Neoehrlichia mikurensis*
*I. acuminatus*	U	*burgdorferi sensu lato*			C. burnetii* F. tularensis*	*Bhanja*	
*I. hexagonus*	V	***burgdorferi sensu lato***				TBEV*	
	U	burgdorferi sensu stricto*, valaisiana*, spielmanii*, garinii*, afzelii*, ***lusitaniae***, bavariensis*	microti*, bovis	conorii*, ***helvetica****, ***sp.**	A. phagocytophilum*	Erve*	
*I. arboricola*	U	garinii*, valaisiana* afzelii*, burgdorferi sensu lato, spielmanii*		*helvetica, *sp.		TBEV*	
*I. lividus*	U	burgdorferi sensu stricto*, garinii*		*(heilongjiangensis), *sp.	C. burnetii*	TBEV* Kama	
*I. canisuga*	U	*burgdorferi sensu lato*				TBEV*	*Yersinia pestis *(*Plague*) *
*I. trianguliceps*	V		microti*				
	U	burgdorferi sensu lato, afzelii*, garinii*			C. burnetii*, F. tularensis* A. phagocytophilum*	Louping-ill*, TBEV*, CCHF*	
*I. vespertilionis*	U					Kul, TBEV*	
*D. reticulatus*	V		microti*	sibirica*	C. burnetii*	OHF*	
	U	*burgdorferi sensu lato*	divergens* bigemina	slovaka*, canada*, conorii*, sp, helvetica*, raoultii*	F. tularensis* Francisella-like A. phagocytophilum*	TBEV*	Bartonella henselae*
*R. sanguineus*	V			conorii*		CCHF*	
	U	*burgdorferi sensu lato*	microti-like*, gibsoni	*Candidatus rickettsia kulagini, *massiliae*, canis, felis*, rickettsi*, rhipicephali,	C. burnetii*, A. phagocytophilum*	Thogoto*	*Ehrlichia canis, Ehrlichia ewingii Hepatozoon canis, Salmonella bacteria, Batonella vinsonii, Rangelia vitalii, Dipetalonema dracunculoides, Mycoplasma haemocanis, Leishmania infantum*
*A. reflexus*	U				C. burnetii*	WNV*, TBEV*, NYMV, QRFV	
*A. vespertilionis*	U	*burgdorferi sensu lato, borrelia sp*			C. burnetii*	rabies*, IK (KTR),SOK	*Wolbachia persica, Treponoma vespertilionis*
*H. aegyptium*	U	*turcica, sp*			C. burnetii*		

*V *confirmed vector, *U *unknown vector status, * recognised pathogen, *WNV *West Nile Virus, *CCHF *Crimean-Congo Hemorrhagic fever, *TBEV *Tick borne Encephalitis virus, *QRFV* Quaranfi Virus, *NYMV *Nyamanini virus, *KTR *Ketera virus, *OHF* Omsk Hemorrhagic fever, *SOK *Sokolok, *IK *Issyk Kul, *KEMV* Kemerovo. In bold pathogen recorded in Belgium. Details in Additional file 3.

### Ecological traits influencing the potential vector role of tick species

#### The major vector *I. ricinus* – a generalist species

**Figure 3:**
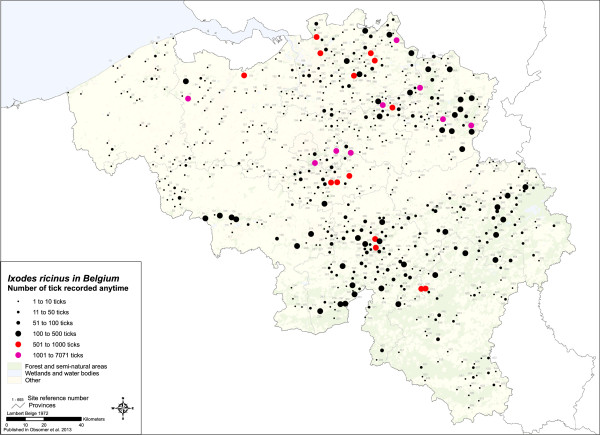
**Map of *I. ricinus *with abundance and hot spots. **Map of existing *I. ricinus* records in Belgium. Abundance is based on number of ticks collected and is not standardized between sites. Collection lasted days in some sites and only a few hours in other sites, however, details on collection methods are often lacking and raw numbers of collected ticks are thus presented. Numbers labelling each site correspond to site number in Additional file 1.

*I. ricinus* is a confirmed vector for many human pathogens (Table 5). Several factors contribute to the efficiency of *I. ricinus*[55]: (1) it is the most abundant tick species in Europe with all stages readily biting humans [56]. (2) This species takes one meal on 3 different hosts in one life cycle and parasitizes a wide range of hosts from mammals (*e.g.* squirrels, badgers, wild boars, cervids, foxes, dogs, cats, cows, rodents, bats) to birds (common blackbird, European robin, pheasant, nest of the Eagle owl), reptiles, lizards and amphibians [57-59]. Larvae and nymphs are found on any hosts and frequently on rodents or birds [60,61]. Adults feed on larger mammals. A large range of hosts means that there are opportunities to encounter many pathogens and disperse in many habitats, with for example *I. ricinus* representing 20% of ticks found on *Parus major*[7]. (3) Phenologic plasticity is wide with all stages surviving 2 weeks under water, several ticks surviving at -10°C, and non engorged nymphs kept alive several months in a fridge (5°C) waking up in minutes if heated by hand contact. The species is vulnerable to desiccation and will not be encountered in arid or non-vegetated environments. Temperatures over 35°C for two weeks will kill all stages [35]. Tolerance to desiccation can be higher in ticks infected by pathogens [62]. This species walks on average 40 cm around its questing post and finds shelter in litter or soil cracks [35]. While ticks interrupt questing to move down the vegetation, in order to rehydrate (quiescence), Perret *et al.* discovered that after dark, quiescence was often interrupted by walking events not necessarily leading to questing, with ticks walking repeatedly 9 m. They suggested that some of these movements represent horizontal walks if the ticks were not confined to their experimental vertical channels and that some of these movements represent activities that enable ticks to find a favourable questing site in nature [63]. Similar experiments but in horizontal arenas did not record such movements [64]. *I. ricinus* is widely distributed in Belgium with sites of apparently higher occurrence (around sites 219, 286, 288, 374, 499) (Figure 3). Lack of standardisation impedes calculation of tick densities and tick abundance and is presented here as the total number of specimens collected in a site. While abundance is expected to decrease from East to West with decreasing forest cover, this is not what comes out of the general map. Few occurrences are recorded in the Southeast despite high forest cover, but few surveys are carried out in that region, while many surveys took place in Campine to clarify tick presence. The large abundance around the Meuse area is picked up as well as decreased abundance between the Meuse and Brussels, which corresponds to a highly cultivated area (Hesbaye). Abundance is also lower along the coast, which is intensely cultivated. High abundances are recorded along the Dutch border and might reflect an increase in tick populations such as recorded in the Netherlands. These trends need, however, to be confirmed because of the lack of standardisation in sampling.

#### Secondary vectors *I. hexagonus* and *D. reticulatus*

*I. hexagonus* is a confirmed vector of *B. burgdorferi s.l.*[65] with 28% prevalence recorded [66]. It is a nidiculous species found in burrows and occasionally in caves [44]. This reduces human vector contact but several characteristics must be highlighted: 1) human infestations were frequent during the war when people sheltered in underground sites during air raids [45] and *I. hexagonus* is considered a common parasite of man in Germany and the United Kingdom [43], 2) the species has a wide range of hosts such as hedgehogs, mustelids, foxes, polecat, badger, roe deer [30], as well as dogs and cats which can increase the spread of ticks and import ticks in gardens close to humans, 3) *I. hexagonus* is found in urban gardens [33], 4) most hedgehogs are infested [67] and the presence of ticks in their surface nests is a potential threat when gardening [33], 5) This species brings pathogens to people normally not at risk for tick bites (just gardening) and can remain undetected for a longer time, 6) it is active throughout the year [68], 7) because of *B. burgdorferi s.l.* transovarial transmission [65], this species could maintain a silent high rate of infection creating long term foci of high infection in the wildlife in areas where it acts as reservoir, 8) finally, Lyme borreliosis could be picked up by *I. ricinus* sharing the same host. In Belgium, *I. hexagonus* is widespread on cats and dogs [13] and has been observed on many mammals (Table 3), on humans [27], in nests, burrows, caves (sites 594, 617), house (site 200) and on grass (sites 492, 670). Populations might fluctuate between years [27]. The species in Belgium carries all the pathogenic species of *Borrelia burgdorferi s.l*., *A. phagocytophilum* as well as the suspected pathogen *R. helvetica*[13].

*D. reticulatus* might be restricted to limited areas and not actively questing in the warm months when people are entering risky areas. *D. reticulatus* is reported on wild boar, cervids, dogs, horses and cats in the French Ardennes [37], wolves or rarely birds [27,32,43]. It can bite humans [69]. Adults are captured by flagging but immatures are nidiculous. This species seems to be expanding its distribution in Europe. In Belgium, the tick has probably been present for some time with one specimen recorded on vegetation in 1950 (site 553), on a dog (site 567) and a human in the eighties (site 376). Established populations have only recently been monitored. Between 2007 and 2009, 16 out of 2297 ticks taken from 161 wild cervids [23] (WILDSCREEN collection) were *D. reticulatus.* From 2010 until March 2012, 150 additional *D. reticulatus* were discovered on 3 cervids in 6 sites including one confirmed by flagging (site 797). Confirmation of the species at a molecular level was carried out when sequences of the 646 bp of a part of the ITS2 gene were successfully obtained for 16 *D. reticulatus*. These 6 sites are in the Southeast but this tick is found on vegetation in the North (60, 130, 172, 535) [34] and on hosts in other locations [13].

#### Ticks parasite of birds

Birds probably carry ticks to most geographical locations but this does not mean that ticks will survive in these locations. The presence of all pathogenic species of *B. burgdorferi s.l.* in *I. frontalis* and *I. arboricola* in Belgium suggests a potential role in the *Borrelia* life cycle [7]. Up to 50% of *I. frontalis*[69] and also *I. lividus*[70] were infected with *B. burgdorferi s.l*. in other countries. While seldom reported as biting humans, they might maintain the pathogen cycle in wildlife*. I. frontalis* (previously *I. pari*) is associated with a broad range of songbirds, including thrushes (Turdidae), the Great tit and the collared dove [7,28,43], with up to 30 specimens per bird [71]. *I. frontalis* is occasionally collected by flagging [72] and evidence suggests that it might be more often present in under-storey vegetation than in nests [7]. In Belgium it has been found on birds in 11 sites. *I. arboricola* is found mainly in Europe but was recorded in Egypt on birds coming back to Europe [27]. It is a nidicolous tick infesting birds and bats [73]. The Great tit might be the dominant host but heavy infestations occur on the Common starling and Peregrine falcon [32]. In Belgium it was recorded in 10 places including 3 sites with 70 specimens (sites 233, 252, 337) where the species was actively surveyed. Specimens were found on birds (Table 3) and in nests of the House martin. *I. lividus* is found on the Sand martin and in their nests [27]. Experimental records showed that these ticks were collected on the Great tit [74] and it was found repeatedly in nests of the House martin in Japan [75].

#### Ticks parasite of rodents and small mammals

Small mammals and particularly rodents are reservoirs of many diseases, but few studies have targeted ticks on rodents in Belgium. Next to *I. ricinus* and *I. hexagonus*, ticks present on rodents and small mammals include *I. canisuga, I. trianguliceps* and *I. acuminatus.* These species are carriers of some pathogens (Table 5) including *B. burgdorferi s.l.*[76-78] with 30% prevalence for *I. canisuga* in Spain [66]. *I. canisuga* is part of a group of species difficult to discriminate morphologically (including *I. hexagonus*, *I. arboricola* and *I. lividus*) [27]. *I. canisuga* is widely distributed and found on half of the foxes in Northern France [30] but also on polecats, weasels, badgers, Eurasian owls, dogs and cats [79,80] with up to 200 specimens reported on one dog [81]. In Great Britain, 11% of the ticks found on dogs were *I. canisuga*[80] but none were reported in a Belgian survey [13]. This nidiculous species is found in nests, burrows or rarely in caves [44] with fed females climbing upwards in crevices above ground [28]. In Belgium, only two specimens were found, on a polecat (site 132) and a nest (site 207). *I. trianguliceps* is found almost exclusively on micromammals including shrews and rodents, exceptionally on moles, birds or goats and very rarely on humans [27,55]. The tick is nidiculous but may wait for hosts on the soil surface [82]. It is commonly found in wet biotopes including moorlands, meadows, or pine, deciduous and birch forests [83]. In France, immature are found with immature of *D. reticulatus*, *I. acuminatus*, and *I. ricinus* on the same rodents [84,85]. In Belgium, *I. trianguliceps* is probably frequent and found on rodents in 8 sites. *I. acuminatus* parasitizes small mammals, birds and sometimes humans and is mostly found in nests and burrows [28]. Only one specimen was found in Belgium on a wood mouse (site 630).

#### Other tick species sharing habitats with humans

Three tick species are not frequent but bite humans and can establish populations inside houses, making them a potential threat. *Rhipicephalus sanguineus* is a tropical tick imported on dogs or rarely with hares, cattles, horses, or plants [55]. The tick cannot survive outside but multiplies inside houses and dog kennels [27]. Ticks hunt for hosts by moving actively [86], can drink free water and survive for years inside. This was shown to cause house infestations in 12 cities in the Netherlands, in Switzerland [87] and 334 foci in Berlin [88]. Tick populations can build up in some years from one engorged female to thousands of ticks and eggs [87,89,90]. Eggs are hidden in cracks and crevices and ticks crawl around. Mean temperature probably limits its northward distribution [91]. This species is rarely found on humans [27], but seems to more willingly bite humans occasionally, representing at times up to 7% of ticks biting men [92]. Up to 22 ticks on one man were reported in France [93]. Some studies suggest that this highlights a change of behaviour related to temperature increase [94,95] but this needs to be further investigated. *R. sanguineus* is a vector of highly pathogenic *Rickettsiae conorii* with confirmed transovarial passage [96]. Prevalence in nymphs infesting houses can reach up to 40% [87], against usually 1% [94] outside. In Belgium, *R. sanguineus* was found in houses in Antwerp [27] (site 210), Hoboken (site 199) [90] and Maldegem (site 71), but also on dogs with a travel history (e.g. sites 338, 426, 536, 684) [13] and on humans (site 210) [27].

*Argas reflexus* is frequent mainly on pigeons but readily bites humans, chickens or horses in buildings in the absence of pigeons [43,55]. With *Argas vespertilionis* (see below) this species belongs to the Argasidae family, which differs from Ixodidae in their feeding habits. Ticks take many short meals. Nymphs and adults engorge in less than an hour. Ticks feed on eight to twelve hosts per life cycle and spend most of their life in the hosts’ habitat [97]. Building infestations are undetected for years because of nocturnal host-seeking behaviour, high host specificity and short meals [98]. However, when pigeons are eradicated, *A. reflexus* appear seeking for alternative hosts [97]. Even if the build-up of a large number of ticks takes years, thousands of ticks were found repeatedly when investigating 188 infested buildings in Germany [98]. Searching for ticks before renovating is now a current recommendation in Germany. Particularities include a long lifetime of up to 9 years without food, low water loss rate, absorption of water vapour at 75% relative humidity and high tolerance of temperature extremes. Movements are restricted to periods of host-seeking, the remaining time being spent resting aggregated in cracks of walls [97-99]. In Belgium ticks were recorded in 7 sites from pigeons, pigeon houses, houses and flats. The bites may cause allergy, anaphylactic shock or loss of consciousness [100]. It is an unconfirmed vector of human pathogens.

*Argas vespertilionis* parasitizes bats [43]. They stay the whole year in or near caves or other shelters (roofs of houses), the hosts being present or not. Eggs were found together with eggs of *I. hexagonus* and *I. vespertilionis*[43,44]. Ticks bite alternative hosts in the absence of their usual host and readily bite man. People reported being bitten in caves or in their bed when bats are in the attics [55]. Larvae attach for 19 days. Nymphs and adults feed in less than one hour [97]. They take many small meals on many hosts potentially accumulating pathogens. *A. vespertilionis* causes irritating bites on humans and viable strains of *C. burnetii* were isolated from ticks which had been dead for a year [97]. While 84% of museum specimens tested positive for *B. burgdorferi s.l.* in the UK [78], this might reflect sample contamination. *Borrelia* sp. organisms related to *Borrelia recurrentis*, *B. duttonii* and *B. crocidurae* were present in numbers in a dying bat in the UK [101] parasitized by *A. vespertilionis*. In Belgium, 11 ticks were found on hosts in four locations.

#### Species of little interest for human health

*Ixodes vespertilionis* was never recorded biting man. It parasites the Lesser horseshoe and the Great bats. It actively searches for hosts by walking slowly on very long legs in caves. It is restricted to the darkest part of the caves, offering 100% humidity [28,102]. Decrease of humidity to 61% increases tick activity until they die after a few hours. All stages are mainly found in caves, moving away with the host but coming back for molting and egg laying [102]. Specimens are not on the ground but on walls and roof crevices. In West France low densities are present in most caves. Ticks were found in caves or on bats in 10 sites in East Belgium [27].

## Conclusion

By providing a countrywide disaggregated approach on tick-borne diseases, this study provides essential preliminary information to build up a national public health policy based on spatial surveillance of tick-borne diseases [103] and help to delineate priorities for future investigations. These are summarised in Table 6. (1) Regarding tick presence, this study highlights the lack of information about current species, and the need to search for species present in bordering regions. (2) In terms of tick distribution, occurrence data offers a useful picture, and this, particularly if the lack of detailed distribution of major determinants (hosts, predators) impeded building of adequate distribution models. However, modelling tick distribution could provide information in places not surveyed, clarify ecology and attach local probabilities to tick presence [104]. For modelling exophilic tick distribution (*I. ricinus* and *D. reticulatus*)*,* examples in the field of malaria vectors show that occurrences can prove useful to delineate species distribution [105]. However, models perform better when explanatory variables englobe most ecologically relevant parameters [106]. Because of their influence on ticks and on the pathogens presence and abundance, distribution of relevant host species or valuable proxies [3] should be integrated in the models with major habitat characteristics. Distribution of nidiculous species could be approached by mapping their specific hosts. (3) Because of the above, the potential roles (hosts, pathogen reservoir versus accidental host [107], influencing tick abundance, influencing pathogen prevalence, and predators) of the birds, mammals, reptiles, amphibian or other local species should be assessed and their distribution investigated. (4) With regards to pathogens, their presence needs to be clarified. The list of micro-organisms/tick associations provided here needs to be narrowed by investigating pathogenicity, identifying reservoirs (ticks and other species), selecting pathogens for which reservoirs are present, and identifying vector capacity for associated ticks. (5) With regards to pathogen distribution, pathogens should be monitored in ticks, reservoirs or any hosts to provide a spatial distribution per pathogen. (6) Human vector contact could be approached using public knowledge from groups at risk, such as nature lovers, hunters, foresters, veterinarians, health practitioners or even the general public. Such facilities for recording tick bites were developed successfully in the Netherlands [9]. (7) Finally a risk map should provide the following locally: integration of the above distribution maps to determine the probability of being bitten by a tick, to obtain an infected tick bite per pathogen, and a list of potential pathogens locally. In this context, potential hotspots could be identified as well as places needing further investigation. This would provide a spatial disaggregation of risk and prepare analysis of seasonal or yearly variations.

**Table 6:** Main findings and suggestions for further research priorities

	**Current knowledge**	**Suggestions for future research priorities**
Tick presence	• The current national list of occurring tick species (not previously available)	• Search for tick species recorded in neighbouring countries country (targeting prefered host species or habitat)
Tick distribution	• A first distribution map for *Ixodes ricinus *based on occurrences which highlights presence of the species in all the provinces	• Build up a distribution model for exophilic species such as *Ixodes ricinus *and *Dermacentor reticulatus *based on habitat preferences and distribution of other influencing species
	• Current very partial knowledge of distribution for the other tick species	• Build up a distribution model for nidiculous species based on distribution of major host species
		• Perform a systematic tick survey across the country
Tick hosts/ reservoirs	• Provide for each tick a list of hosts on which they have been recorded in the country	• For each local vertebrate species check potential host status for each tick species or potential influence on tick population
		• Map the distribution of relevant species
Presence of pathogens	• Potential presence of pathogens such as *Borrelia burgdoferi s.l. *in many tick species	• Check the pathogenicity of each micro-organisms species
	• List of microorganisms potentially present locally or aborad in local ticks species	• For pathogenic microorganisms check vector status of associated ticks Identify presence of potential reservoirs for pathogens (tick/ hosts)
	• List of tick/ micro-organisms associations	• Investigate pathogen distribution across species to better comprehend
		• risk before modelling risk map
Pathogen distribution	• First map of (suspected) pathogens found in ticks	• Search for additional pathogens in ticks of the country
		• Make a pathogen distribution map (found in ticks, hosts, reservoirs)
Human-vector contact		• Use public knowledge from nature defense group, scouts, veterinary, general practitioner to localise and quantify tick bites
Tick-borne diseases risk map	• Some hot spot with highest *I. ricinus *abundance are highlighted but because of unreliable sampling those should be further investigated	• Investigate presence and prevalence of pathogenic species
		• Make a countrywide standardised survey to allow comparing abundance between sites.
		• In a given area, what is the probability 1) to get a tick bite, 2) that this tick was infected with pathogens 3) infected by which pathogen(s)

*I. ricinus* is the main vector of diseases in Belgium because it is present in most vegetated areas, carries many pathogens and is responsible for most tick bites in humans. Its distribution highlights the possibility of becoming infected by the pathogenic agent of Lyme borreliosis in any province. Other species might play a role by maintaining pathogens present in wildlife, or by bringing pathogens closer to people in their houses and gardens. While the number of bites on humans caused by other species is less than by *I. ricinus*, occurrence of such an event in unexpected areas such as houses or gardens or during the winter season extends the risk to people not considered to be at risk and increases the probability of a tick bite being ignored. Some places seem to pose a greater risk with more abundant tick populations, higher diversity of pathogens or both, but this should be confirmed. Current prevention measures target *B. burgdorferi s.l.* mainly through deticking. Other pathogens are increasingly investigated and found in domestic animals, wildlife or humans and transmission of some of those can occur at the time of the tick bite without delay (e.g. TBEV). Avoidance behaviour should be promoted such as avoiding areas with ferns [108], wearing boots and long trousers or repellent, using deticking as the second line measure. Another reason to avoid ticks is that in Belgium only 30% of Lyme patients remember a bite and 35% never develop *Erythema migrans*[109]. Lyme disease can be difficult to diagnose in the absence of *Erythema migrans* and particularly if additional symptoms are caused by co-occurring pathogens.

The analysis of the presence of pathogens in ticks might be easier than in human blood as organisms are more easily detected by PCR in ticks than in blood. Systematic surveys using ticks as sentinels could assess the prevalence of the pathogens in wildlife. Geolocation of tick and pathogen records allows integration into a more general databases such as those developed in the framework of The Vbornet [5] or the TICK MAP initiative [110] and could help fulfil the empty maps for tick and tick-borne disease occurrence in Belgium [4,6]. This study based on imperfect sampling data calls for an increased surveillance of ticks and tick-borne species at a detailed spatial scale as well as clarification of local vector status for tick species and pathogens which occur in these species. A systematic survey of ticks and associated pathogens is called for in Europe, as well as better characterisation of species interaction in the ecology of tick-borne diseases.

## Abbreviations

ARSIA: Association Régionale de Santé et d’Identification Animale; BLAST: Basic Local Alignment Search Tool; CCHFV: Crimean Congo Hemorrhagic Fever virus; DGZ: Dierengezonheidzorg Animal Health Care Flanders Belgium; DNA: deoxyribonucleic acid; dNTP: deoxy ribonucleotide triphosphate; IK: Issuk Kul virus; IRSIA: Institut pour l′encouragement de la recherche dans l′industrie et l′agriculture; IRSNB: Institut Royal des Sciences Naturelles de Belgique; ITS2: second Internal Transcribed Spacer 2; LIV: Looping Ill virus; OHFV: Omsk Hemorragic fever virus; PCR: Polymerase Chain Reaction; RLVBD: Reference Laboratory for Vector Borne Disease; SOK: Sokuluk virus; TBEV: Tick-borne Encephalitis virus; UA: University of Antwerp collection; UGENT FOREST: Ghent University, Forestry department collection; UGENT VETE: Ghent University, Faculty of Veterinary Medicine; UCLIREC: Université Catholique de Louvain Clinical and experimental research institute; WILDSCREEN: Network for disease surveillance in wildlife; WNV: West Nyle virus.

## Competing interests

The authors declare that they have no competing interests.

## Authors’ contributions

VO: Design of the study, compilation of the record, writing up of the manuscript. MW: Organisation of data collection, tick and pathogens identification, writing up the manuscript. AL: Organisation of data collection, revising the manuscript. EC: Details on data collection, revising the manuscript. PH: Organisation of data collection, tick and pathogens identification, revising the manuscript. DH: Organisation of data collection, tick and pathogens identification, writing up the manuscript. MM: Organisation of data collection, tick and pathogens identification, revising the manuscript. JM: Organisation of data collection, revising the manuscript. ML: Details on data collection, revising the manuscript. WT: Details on data collection, revising the manuscript. LL: Details on data collection, tick and pathogens identification, revising the manuscript. TH: tick and pathogens identification, revising the manuscript. GvI: Collected many data, tick identification, advice on manuscript design, provided expert knowledge, and revised the manuscript. All authors read and approved the final version of the manuscript.

## Supplementary Material

Additional file 1 **Detailed information available per sites of occurrence for ticks recorded in Belgium.** Description: table per site with geographical coordinates in latitude-longitude and in Belgian Lambert, list of records included in the site, list of species included in the sites, literature/collection references for the sites and general quality level. Additionally to the main tab a Memo tab describes each field. Click here for file

Additional file 2 **Records of tick occurrence in Belgium. **Description: a kmz file to open in Google Earth with 5 folders to navigate into the database. (1) Records per tick species: one point per record classified in folders by tick species, (2) Records per pathogens: one point per record classified in folders by pathogen genus then species, (3) Records per authors: one point per record classified in folders by authors, (4) Sites: one point per site with information on tick species and pathogens, (5) Records: One point per record with information available in the Additional file 1 included. Click here for file

Additional file 3 **Micro-organisms found locally or abroad in tick species present or potentially present in Belgium. **Description: tick species, reference of the article and details on the records, discussion on pathogenicity to humans and potential transmission by tick species, and pathogenicity status as well as reference to specific articles and overall assessment of vector status. Click here for file
